# Removal of direct dyes from wastewater using chitosan and polyacrylamide blends

**DOI:** 10.1038/s41598-023-42960-y

**Published:** 2023-09-21

**Authors:** Medhat M. H. Elzahar, M. Bassyouni

**Affiliations:** 1 Department of Civil Engineering, Faculty of Engineering, Port Said University, Port Fouad, Port Said, 42526 Egypt; 2 Department of Chemical Engineering, Faculty of Engineering, Port Said University, Port Fouad, Port Said, 42526 Egypt; 3 Center of Excellence in Membrane-Based Water Desalination Technology for Testing and Characterization, Port Said University, Port Said, 42526 Egypt; 4 East Port Said University of Technology, North Sinai, Port Said, 45632 Egypt

**Keywords:** Engineering, Chemical engineering, Environmental sciences, Environmental chemistry, Pollution remediation

## Abstract

This study investigated the feasibility of employing neat chitosan powder, polyacrylamide, and chitosan micro-beads as adsorbents for the rapid and efficient removal of Direct Blue 78 dye from textile industrial wastewater. A series of batch experiments were conducted to examine the impact of adsorbent dose, contact time, and pH on the adsorption process. The physicochemical analysis, including FTIR, zeta potential analysis, and SEM were performed to identify the adsorption mechanism of chitosan powder and micro-beads. It was found that increasing the powder chitosan dose to 4.5 g/L and contact time up to 40 min resulted in achieving a significant increase in dye removal efficiency up to 94%. The highest removal efficiency of 94.2% was achieved at an initial dye concentration of 50 mg/L, a chitosan dosage of 4.5 g/L, and an optimized contact time of 60 min. Utilizing a polyacrylamide gel dose of 45 mL/L reduced the sedimentation time of chitosan from 8 h to 5 min. Equilibrium studies showed an initial L-shaped equilibrium curve, indicating that the adsorption process primarily arises from electrostatic interactions between dye molecules and adsorbent particles (physical forces). The Langmuir isothermal model demonstrated the best fit to the equilibrium data. Combining chitosan powder with polyacrylamide gel emerges as an economically viable choice for dye removal in industrial wastewater effluents, offering a cost-effective alternative to pricey commercial adsorbents. The results of the study revealed that the presence of polyacrylamide dye enhanced the removal efficiency and settling time of DB78 dye using chitosan.

## Introduction

As the global population approaches nine billion, preserving and treating water has become a top priority due to urbanization, new consumption habits, and climate change. By 2030, nearly half of all people may face water stress, and if current trends continue, North America and Sub-Saharan Africa's annual water demand might increase by 42% and 283%, respectively, over 2005 levels^1,2^. To address this issue, the 2030 Agenda for Sustainable Development aims to incorporate water-related issues into all 17 goals and make a specific commitment to ensuring the availability and sustainable management of water and sanitation for all^2^. The textile industry is a major contributor to liquid waste, with ~ 7 × 10^5^ tons of dyes produced worldwide annually, some of which are released into the water during the fabric dying process These released dyes reach 20% of the total direct dyes applied quantity^3,4^. The wastewater from the textile industry contains a high concentration of complex dyes, leading to higher organic carbon values than standard limits if proper treatment methods are not carried out^4,5^.

The complex aromatic structure of synthetic dyes makes them difficult to decompose and separate, leading to their classification as toxic and carcinogenic contaminan ^6^. Fabric dyeing processes use various types of dyes, including anionic, cationic, and non-anionic dyes^7^. Anionic dyes are particularly hazardous because they are not easily degradable and can contaminate the food chain. If these dyes are not properly separated, they could pose a significant threat to public health and the environment^8–11^.

For over a century, various methods and techniques have been utilized to treat wastewater. Numerous processes have been developed and a wide range of techniques and materials have been tested to effectively treat wastewate ^12,13^. Many of these techniques rely on coagulation, flocculation, flotation/sedimentation, filtration, and adsorption at different stages of the treatment system, to remove general contamination and toxic pollutants from wastewater effluent^14,15^. The development of improved treatment processes involves incorporating the latest discoveries in physical, chemical, and biological mechanisms into the optimal technology for wastewater treatment. This is done to conserve water and promote sustainability in the face of increasing global population numbers, which leads to greater water demand and scarcity^6^.

Out of various methods for removing color from wastewater, adsorption is considered the most versatile treatment technology^16,17^. This process should be straightforward, eco-friendly, and able to efficiently extract various types of dye waste at a low operating cost^18^. Activated carbon is a known adsorbent with a relatively safe reputation due to its ability to remove multiple types of hazardous pollutants from water and wastewater effluent^19,20^. However, its high cost makes it less commonly used. As a result, significant efforts have been made to develop both conventional and non-conventional inexpensive adsorbents using various materials such as chitosan, sawdust, municipal solid waste, red mud, zeolites, and sugar industry waste^21^.

Chitosan is a linear copolymer created by deacetylating chitin, the world's second-most abundant biopolymer after cellulose^22^. Due to its distinctive properties, chitosan is utilized in various industrial applications. Additionally, chitosan has garnered significant research interest as a promising coagulant and flocculent due to its environmentally friendly characteristics. Laboratory studies have demonstrated that chitosan-based materials serve as eco-friendly coagulants and flocculants for water and wastewater treatment, thanks to their natural biological properties and biodegradability^23^. Although chitosan has several advantages in wastewater treatment such as high adsorption capacity, biodegradability, low toxicity, antimicrobial properties, floc formation, and renewable resources, its several limitations represented in pH sensitivity, ionic strength, slow kinetics, and high cost usually obstruct its widespread.

Recently, researchers have become increasingly interested in chemically modifying chitosan as a potential adsorbent, as well as investigating the impact of fillers and blends on its removal efficiency to decrease the impact of its limitations^24^. Chitosan functions as a coagulant by removing chlorella sp. from algae turbid water, reducing turbidity in seawater, and harvesting microalgae. Chitosan can be recycled at industrial and commercial levels and functions as a chelating agent for many heavy metals, such as arsenic, molybdenum, cadmium, chromium, lead, and cobalt. Chitosan has been the subject of various investigations for its potential use as a coagulant in drinking water^25^.

In this study, both neat chitosan powder and chitosan micro-beads were utilized to remove Direct Blue 78 dye (DB78) from synthetic wastewater. Chitosan has been extensively studied for its ability to remove dyes from textile wastewater effluent, and the goal of this research is to achieve the highest removal efficiency of DB78 with short contact and settling times. Bench-scale experiments were conducted to examine the effect of adsorbent dose, contact time, and pH. Additionally, the physicochemical characteristics of chitosan powder and chitosan micro-beads were analyzed to determine the optimal experimental conditions for direct dye removal (including coagulant dose, pH, and contact time).

## Materials and methods

### Materials

**Figure 1:**
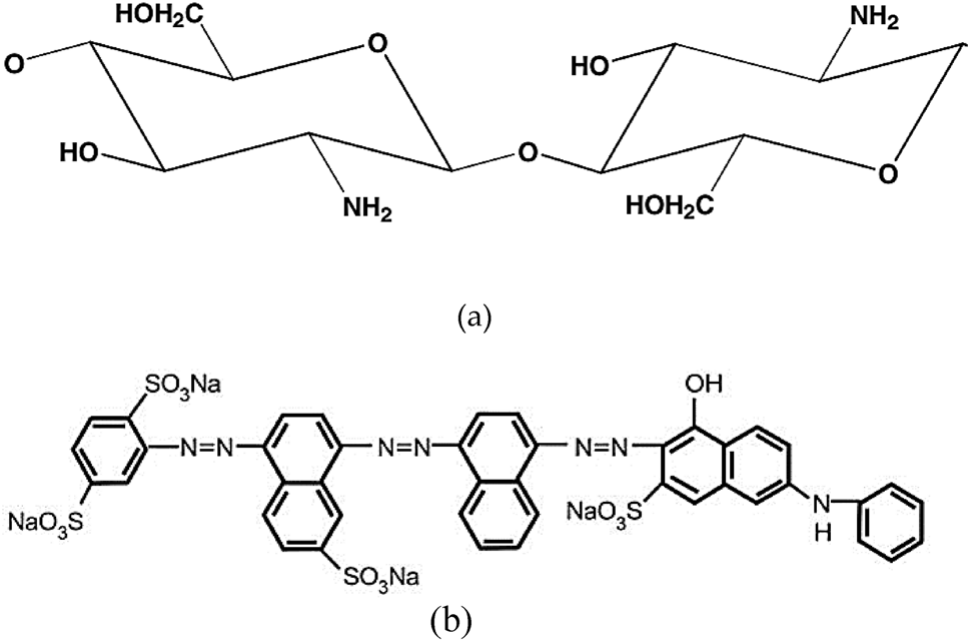
The chemical structure of (**a**) chitosan and (**b**) DB78 dye.

High molecular weight chitosan (Ch) was obtained from Titan Biotech, India. The characteristics of Ch were; a white powder has a molecular weight range from 140 to 220 kDa, degree of deacetylation (DAC) = 82%, viscosity = 36000 cps, and density 0.15 g/mL. The chemical structure of chitosan is shown in Fig. 1a. The polyacrylamide (PAM) was obtained from Sigma-Aldrich, with purity > 99%. The Direct Blue 78 (DB78) with the commercial name Tubantin GLL 300 has a molecular weight of 1059.95 g/mol and a maximum wavelength (λ_max_) of 604 nm. It is soluble up to 10 g/L at 25 °C and is commonly used in the textile industry. Given its prevalence in this industry^23^. Direct Blue 78 is an azo dye commonly used in the textile industry to dye cotton, wool, and silk fabrics. Heterocyclic structures as shown in Fig. 1a are often resistant to biodegradation and can persist in the environment for a long time^26^. The chemical structure of DB78 is depicted in Fig. 1b.

### Preparation of chitosan micro-beads and polyacrylamide gel

The chitosan solution was prepared under magnetic stirring for 4 h by dissolving 1.5 g (1.5 wt%) of chitosan powder into diluted acetic acid (concentration 1% wt./wt.) to form a 100 mL chitosan gel sample. The final prepared gel was dropped into a 0.7 M NaOH solution (contact time 4 h), using a micropipette to form the beads. The formed beads were washed with distilled water. Finally, the beads were oven-dried at 50 °C. The polyacrylamide gel was obtained by dissolving 3 g of anionic polyacrylamide powder into 1000 mL of deionized water.

### Adsorption studies

In this study, a batch adsorption system was utilized for dye removal from synthetic wastewater. The chitosan powder used ranged from 0.5 g to 9 g per 1000 mL of synthetic wastewater, with an initial concentration of DB78 dye at 50 mg/L. The dye was stirred at a speed of 150 rpm for a contact time of up to 70 min at a room temperature of 25 ± 2 °C. Since the chitosan powder had a long settling time, 45 mL of anionic polyacrylamide gel was added to 1000 mL of synthetic wastewater after reaching equilibrium concentration to speed up the sedimentation process. In addition to the chitosan powder, chitosan micro-beads were also used with doses ranging from 0.5 to 15 g/L. The removal efficiency was determined through spectrophotometric analysis, which involved measuring the dye concentration before and after the adsorption process at λ_max_ = 600 nm for DB78. The removal (%) of DB78 dye was calculated using Eq. (1).

1 $$\% \;{\text{Removal}} = \left( {{\text{Ci}} - {\text{Ce}}} \right)*{1}00/{\text{Ci}}$$

where Ci (mg/L) is the initial concentration of the dye (before adsorption) and Ce (mg/L) is the concentration of the dye at the equilibrium state (after adsorption)^27^. In the same way, the adsorption capacity (mg/g) was calculated using Eq. (2).

2 $${\text{q}}_{{\text{e}}} = \left( {{\text{C}}_{{\text{i}}} - {\text{C}}_{{\text{e}}} } \right){\text{V}}/{\text{m}}$$

where q_e_ is the adsorption capacity (mg/g), C_i_ and C_e_ are the initial and equilibrium concentration (mg/l) of dyes respectively, V is the volume (mL) of effluent and m the mass (g) of adsorbent^28^.

### Materials and methods

The chitosan was dispersed in water using a sonicator for 30 min in an ultrasonic bath at 4% (w/v). Model USC-1400 was used at 40 kHz of ultrasonic transducer. The Malvern 3000 Zetasizer NanoZS (Malvern Instruments, UK) was used to determine the particle size. This apparatus measured the diffusion of particles moving under Brownian motion and translated the data to size and size distribution using dynamic light scattering. It also employed laser doppler micro-electrophoresis to provide an electric field to a dispersion of particles, which then moved at a rate proportional to their zeta potential. The Smoluchowski algorithm was used to determine the particle size. Scanning electron microscopy (TESCAN MIRA-High Resolution scanning electron microscope, Tescan Essence Company, Brno, Czech Republic) at 5 keV and an energy dispersive X-ray (EDX) were used to analyze the samples' elements (Oxford instrument nano analysis detector, UK).

The surface area of the chitosan powder and chitosan beads samples were measured in the presence of N_2_ adsorption at -195.65 °C using surface area analyzers (Autosorb-l-C-8, Quantachrome, USA). Before adsorption studies, the samples were degasified at 200 °C for 4 h. By applying the BET (Brunauer–Emmett–Teller) equation to adsorption data, the BET surface area for the sample was determined.

For the materials used, FTIR analyses were carried out utilizing (a VERTEX 80v vacuum FTIR Spectrometer, Bruker Corporation, Germany).

In this study, colorimetric analysis was performed using (LAMOTTE smart spectrophotometer V3 2000-01-MN, USA).

## Results and discussion

### Characterization of adsorbents

Adsorbent samples were characterized using Zeta potential to measure the chitosan particles’ net surface charge. FTIR analysis was conducted to determine the chemical structure of samples and the mechanism of the adsorption process. SEM analysis was applied to investigate the surface morphology of chitosan particles.

### Zeta potential analysis

**Figure 2:**
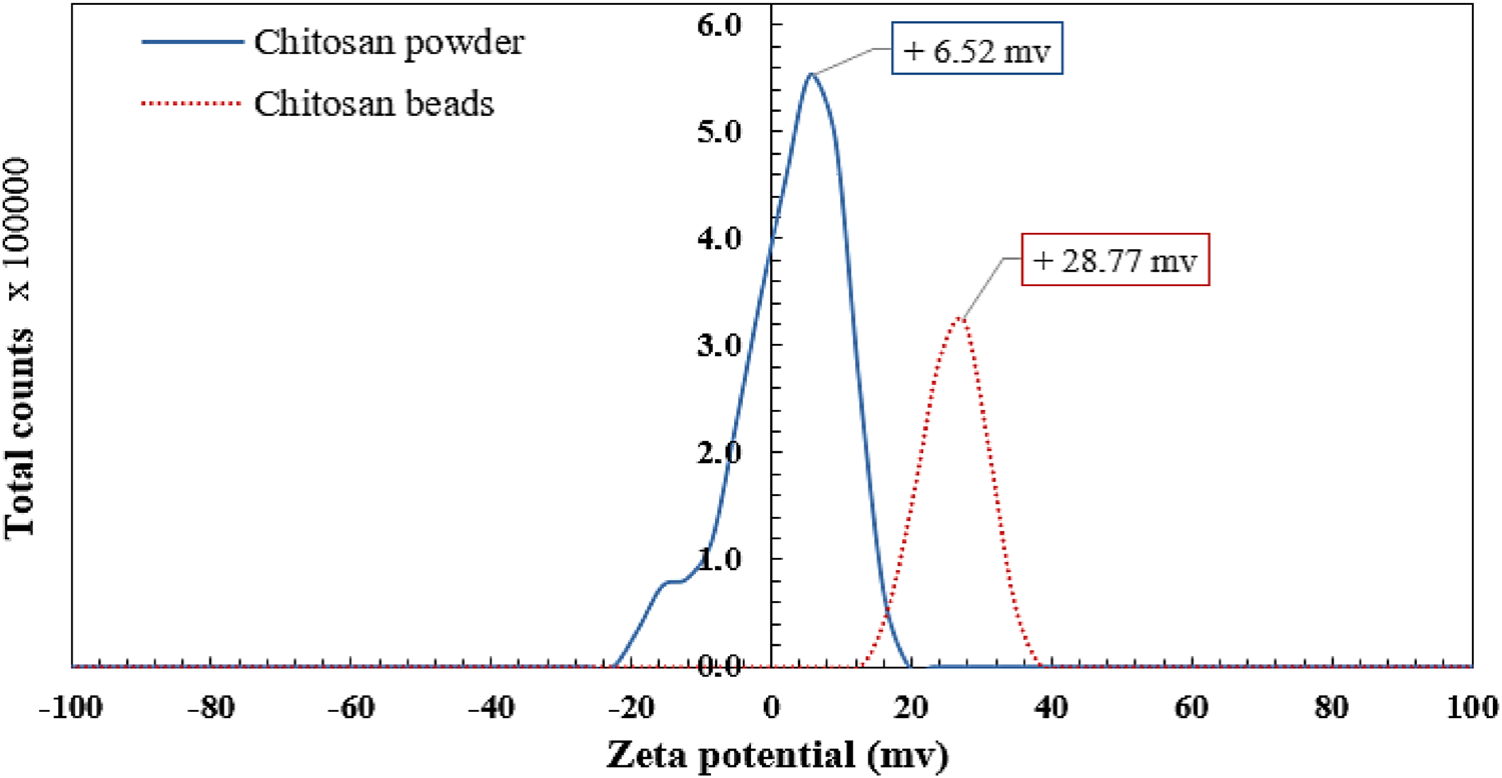
Zeta potential analysis for neat chitosan powder and chitosan micro-beads.

The neat chitosan powder has a net surface charge of + 6.52 mv, according to the zeta potential analysis as shown in Fig. 2. The positive charge of the powder is attributed to the presence of NH_2_ groups on the surface of the chitosan particles. The net surface charge of chitosan micro-beads was + 28.77 mv. The swelling of chitosan powder into chitosan beads under acidic conditions, which protonates the amine groups NH_2_^+^ into NH_3_^+^, causes a larger improvement in microbead surface charge.

### FTIR analysis

**Figure 3:**
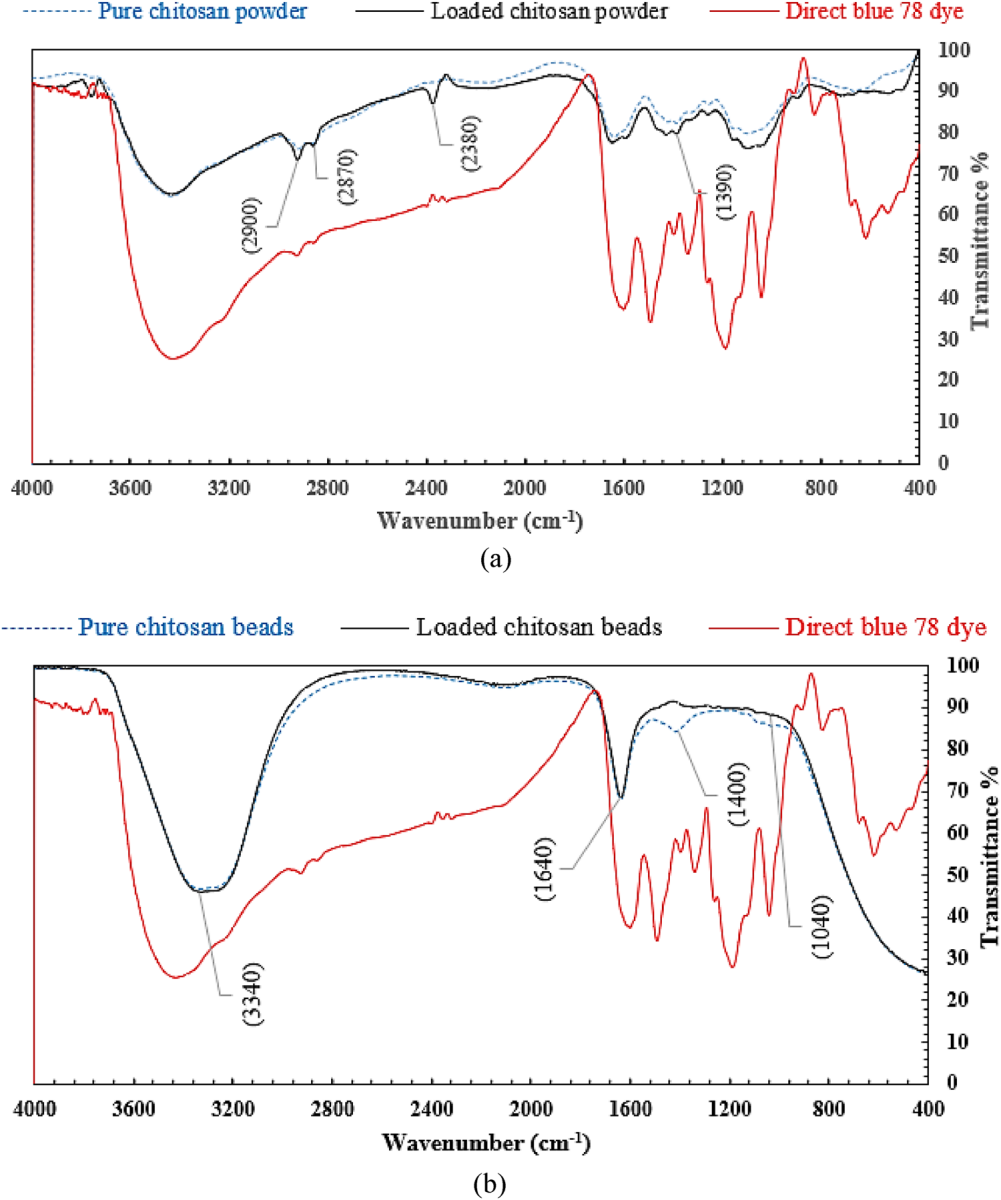
FTIR analysis for (**a**) Neat and loaded chitosan powder and (**b**) Neat and loaded chitosan micro-beads.

The FTIR analysis of neat-loaded chitosan powder and DB78 dye was used to classify the main infrared (IR) bands of organics and determine the adsorption mechanism (physisorption or chemosorption). In Fig. 3a, the peaks at 2900 and 2870 cm^−1^ can be attributed to C–H symmetric and asymmetric stretching respectively. The CH_2_ bending and CH_3_ symmetrical deformations were approved by the presence of a peak at 1390 cm^−1^. The chemical bond formed between dye molecules and –NH_2_ groups on the surface of chitosan particles after the adsorption process (wavenumber 2380 cm^−1^) is attributed to the new peaks found in FTIR spectra for loaded neat chitosan (chemical adsorption). These results were also reported by Fernandes Queiroz et al.^24^. Additionally, the FTIR analysis of loaded neat chitosan and micro-beads are shown in Fig. 3b. It is revealed that there is no significant variation between the beads’ properties before and after the adsorption process (the two spectrums are identical). This means that the anionic dye molecules and NH_3_^+^ groups on the surface of the bead electrostatically interacted to cause the adsorption process to occur (physical adsorption).

### Surface morphology

**Figure 4:**
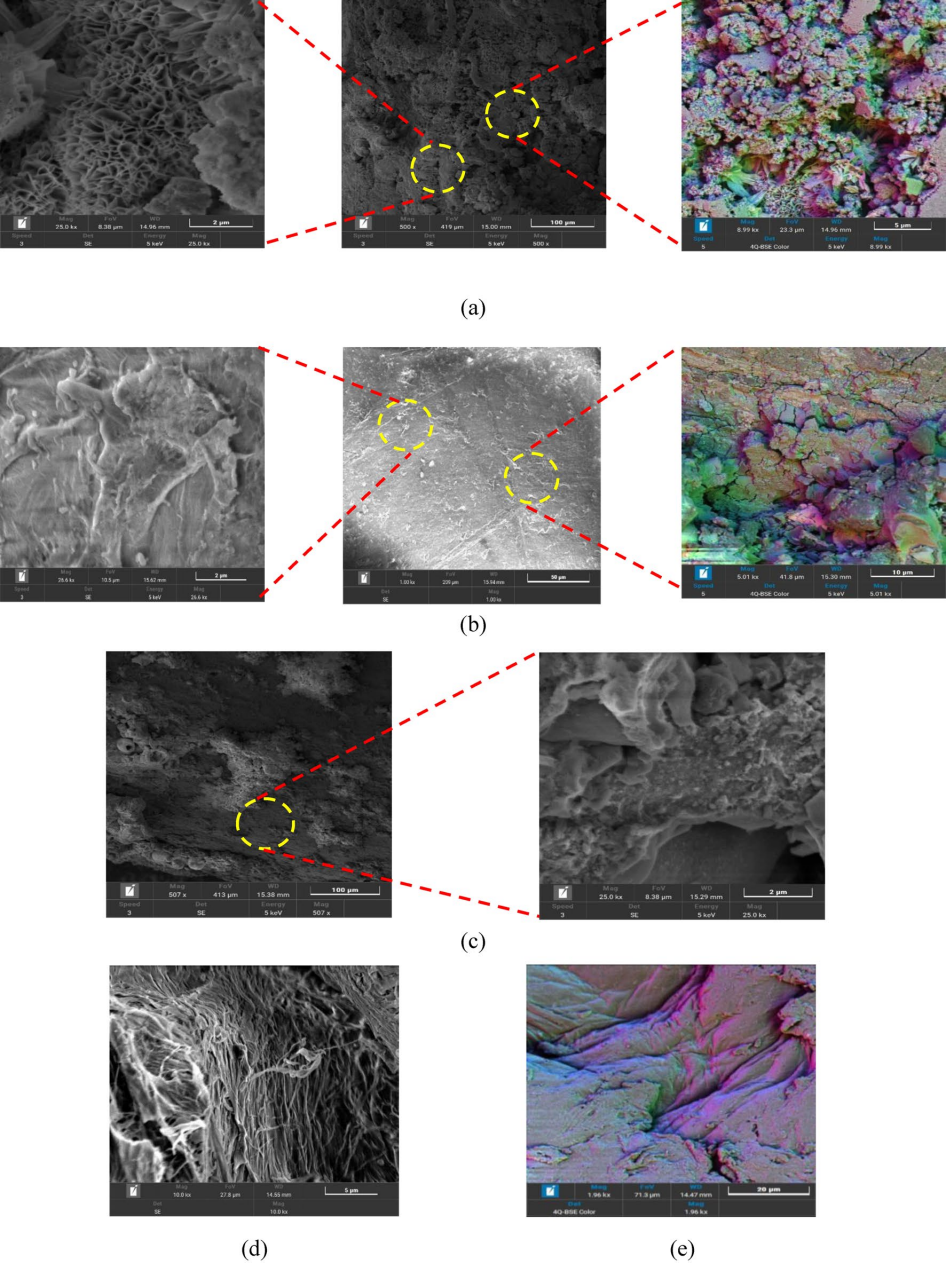
SEM analysis for (**a**) Neat chitosan powder, (**b**) Loaded chitosan powder, (**c**) loaded chitosan powder/ Polyacrylamide gel, (**d**) Neat chitosan bead, and (**e**) Loaded chitosan bead.

To investigate the surface morphology of chitosan particles before and after the adsorption process, the SEM analysis was conducted as shown in Fig. 4. It was observed that the chitosan powder has a rough surface with enhanced porous structure as shown in Fig. 4a. After the adsorption process, the chitosan surface has a significant difference, all the pores in the chitosan particle surface have completely disappeared as a result of its saturation with dye molecules as shown in Fig. 4b. After the adsorption, the polyacrylamide gel was used to accelerate the settling time for loaded chitosan particles. As shown in Fig. 4c, the polyacrylamide gel gathered the fine particles of powdered chitosan to form a large particle with a low settling time.

### BET analysis

The BET surface area analysis was conducted to investigate the effect of the modification process on the adsorption characteristics of chitosan such as surface area and pores volume. The analysis showed that there is a higher difference in surface area and pores volume for chitosan powder and chitosan beads. For chitosan powder, the BET surface area and pores volume were 46.77 m^2^/g, 0.106 cm^3^/g, and the average power size was 1.67 nm. After the modification process (chitosan beads), the BET surface area decreases to reach 18.93 m^2^/g. The results shown in Table 1 indicated that, the applied modification process (converting the chitosan from powder into beads) has a major negative effect on BET surface area and pores volume.

**Table 1:** BET analysis for chitosan powder and chitosan beads.

Parameter	Chitosan powder	Chitosan beads
Average pores radius (nm)	1.67	0.93
BET surface area (m^2^/g)	46.77	18.93
Pores volume (cm^3^/g)	0.106	0.062

## Adsorption tests

### Effect of adsorbent dose on DB78 dye removal efficiency

The equilibrium loading and dye removal percentage are directly related to adsorption processes. As the equilibrium loading increases, it means that more dye molecules are being adsorbed onto the adsorbent surface, resulting in a higher amount of dye removal from the solution. Therefore, a higher equilibrium loading corresponds to a higher dye removal percentage. Efficient adsorbents, such as chitosan, with a higher equilibrium loading, are capable of adsorbing more dye molecules, leading to a more effective dye removal from the solution. Factors that influence the equilibrium loading and, consequently, dye removal percentage include the surface area and porosity of the adsorbent, the concentration of the dye in the solution, the contact time between the adsorbent and the dye solution, and the affinity between the dye molecules and the adsorbent surface.

**Figure 5:**
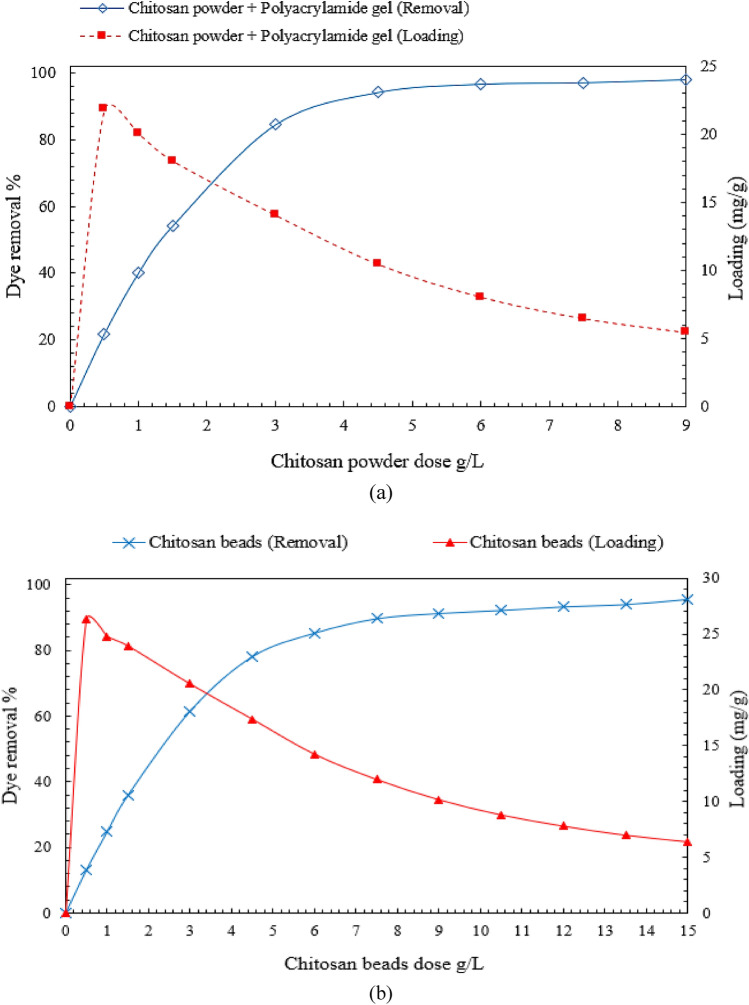
Effect of adsorbent dose on DB78 dye removal efficiency. (**a**) Neat chitosan powder and (**b**) Chitosan micro-beads.

Chitosan powder and chitosan micro-bead concentrations were varied to investigate their effect on direct blue 78 dye removal efficiency. It was found that increasing the adsorbent dose (increasing the adsorption active sites) led to decrease the equilibrium loading while increasing the removal efficiency until it reached the maximum efficiency and then approached a constant value as shown in Fig. 5a, b. Experiments were conducted by varying the loading of chitosan powder from 0.5 to 9 g/L and chitosan micro-beads from 0.5 to 15 g/L on DB78 dye solutions with initial concentrations of 50 mg/L at a fixed temperature of 25 °C, pH (3–4), stirring speed 150 rpm and mixing time 60 min.

Batch adsorption tests of chitosan powder revealed that a removal percentage of 94.2 percent and an equilibrium loading of 10.5 mg/g can be obtained by using a chitosan dose of 4.5 g/L for the solution with an initial concentration of 50 mg/L, as shown in Fig. 5a. The maximum equilibrium loading achieved was 21.85 mg/g, which was obtained by using a chitosan dose of 0.5 g/l in a solution with an initial concentration of 50 mg/l. After the adsorption process was completed and the equilibrium concentration was reached, a 45 mL/100 mL dose of polyacrylamide gel was added to the aqueous solution and mixed for 10 min at a stirring speed of 50 rpm to accelerate the settling time of chitosan particles. The settling time of chitosan particles has been significantly reduced. It was reduced from 8 h (without the use of polyacrylamide gel) to 10 min (using polyacrylamide gel).

Using chitosan beads, the removal percentage of 94.1 percent and an equilibrium loading of 6.9 mg/g were obtained by using a chitosan dose of 13.5 g/L for the solution with an initial concentration of 50 mg/L, as shown in Fig. 5b. The maximum equilibrium loading achieved was 26.3 mg/g, which was obtained by using a chitosan dose of 0.5 g/l in a solution with an initial concentration of 50 mg/L.

The chitosan powder achieved a higher removal efficiency of 94.2 percent using a lower adsorbent dose of 4.5 g/L, while the chitosan micro-beads achieved a removal efficiency of 94.1 percent using a higher adsorbent dose of 13.5 g/L. This significant difference in dose amounts can be attributed to neat chitosan particles having a higher surface area due to their smaller particle size when compared to micro-beads.

### Effect of pH on DB78 dye removal efficiency

**Figure 6:**
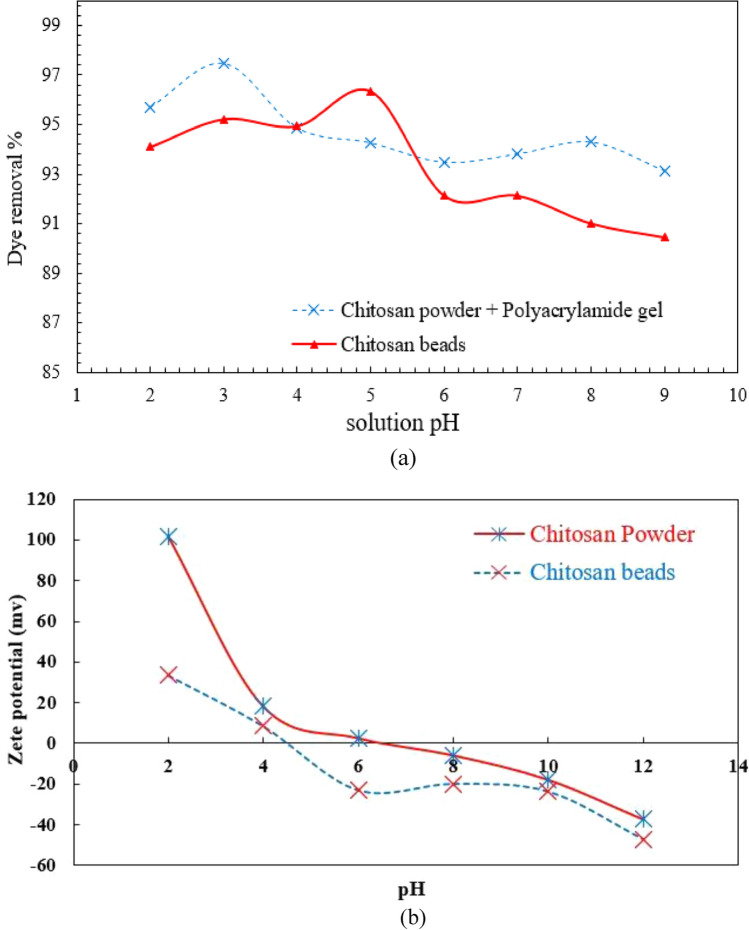
(**a**) Effect of pH on DB78 dye removal efficiency using chitosan powder and chitosan micro-beads and (**b**) Effect of pH on zeta potential of chitosan particles.

The effect of the initial pH for the dye solution was experimentally investigated under a pH range from 2 to 9 and the results can be observed in Fig. 6a. Using chitosan powder, the solution pH has a major effect on chitosan adsorption behavior. It reaches its maximum removal efficiency of 98% under acidic conditions (pH = 3) in comparison with a 93% removal efficiency under alkaline conditions (pH = 9). The experiments were conducted under the following conditions; temperature 25 °C, mixing speed 150 rpm, micro-bead dose 4.5 g/L, and contact time 60 min. Moreover, the chitosan micro-beads reach their maximum removal efficiency of 96.4% under acidic conditions (pH = 4) in comparison with an 80% removal efficiency under alkaline conditions (pH = 4.5). The experiments were conducted under the following conditions; temperature 25 °C, mixing speed 150 rpm, micro-bead dose 13.5 g/L, and contact time 60 min.

The effect of pH on the zeta potential of chitosan particles was also studied as shown in Fig. 6b and the results showed that the points of zero charge (PZC) for chitosan powder and chitosan beads were reached at pH = 6.8 and pH = 4.9. Furthermore, it was observed from Fig. 6a that the maximum removal efficiencies for chitosan powder and chitosan beads were achieved at pH = 3 and pH = 4.5 respectively, when the pH of the aqueous solution is lower than PZC the chitosan particles will have a positive charge on its surface which enable it to adsorb the DB78 dye (anionic dye) by electrostatic interaction.

### Effect of contact time on DB78 dye removal efficiency

**Figure 7:**
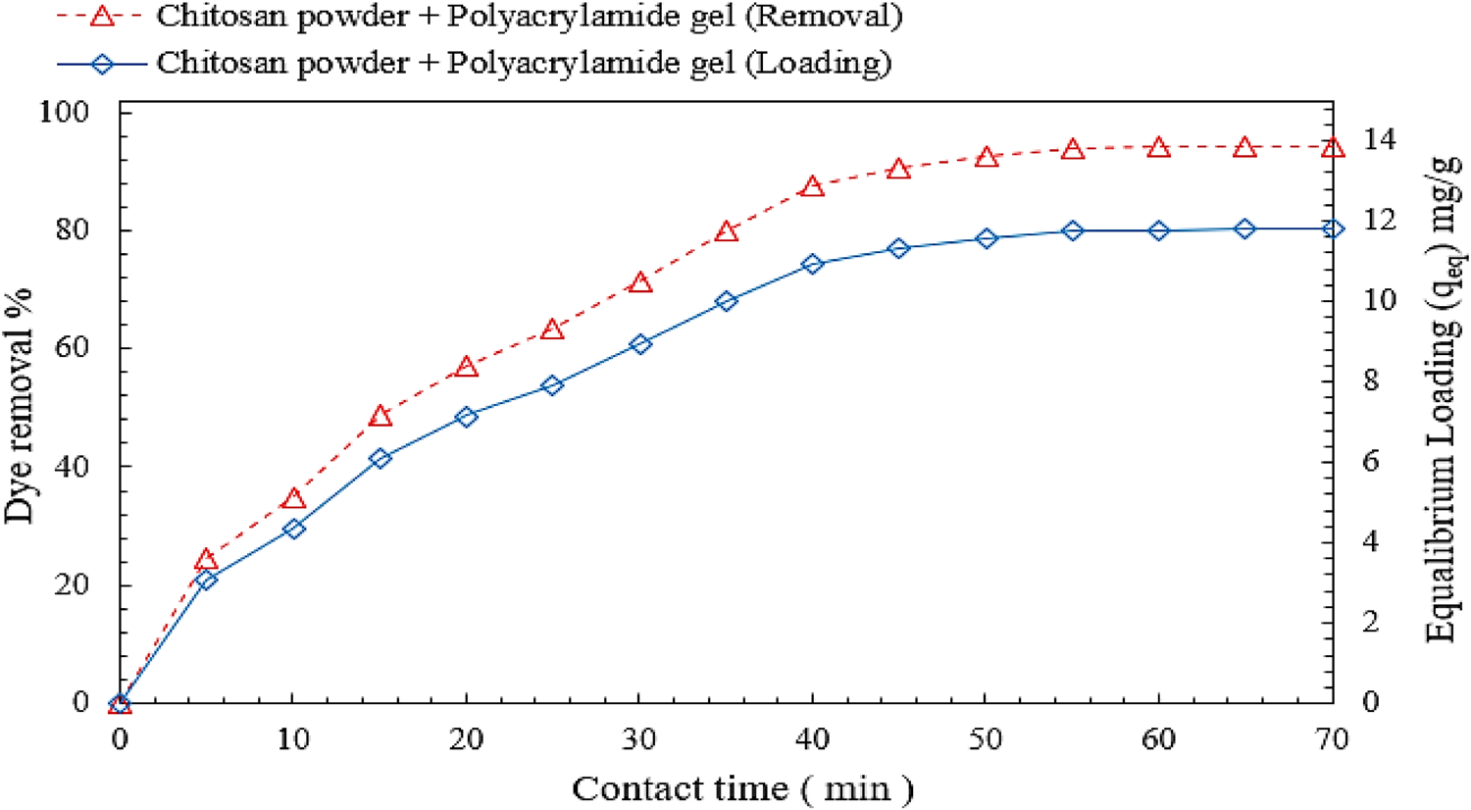
Effect of contact time on DB78 dye removal efficiency and equilibrium loading using chitosan powder.

Chitosan microbead loading (mg/g) and the removal efficiency of DB78 dye were both investigated. According to Fig. 7, the percentage of dye removal increased with increasing mixing time It reached the optimal removal efficiency (equilibrium concentration C_e_), at which point the chitosan powder reached its maximum loading capacity (equilibrium loading q_e_).

For 60 min contact time, 4.5 g/L as a dose of neat chitosan powder, the equilibrium concentration decreased to 2.90 mg/L with a dye removal efficiency of 94% and optimum loading capacity of 11.2 mg/g for an initial dye concentration of 50 mg/L. These experiments were conducted under the following conditions; a temperature of 25 °C, mixing rate of 150 rpm, and contact time range (60) min.

Another study by Saha et al.^29^, examined the impact of chitosan powder dose on the removal of Reactive Black 5 dye from aqueous solutions. The study revealed that increasing the chitosan powder dose resulted in higher dye removal efficiencies. This behavior can be attributed to the availability of more adsorption sites with an increase in chitosan dosage, leading to enhanced interactions between the dye molecules and the adsorbent. Furthermore, the results revealed that the adsorption capacity increased with a decrease in the pH. The maximum adsorption capacity (39.5 mg/g) was reached at pH 5.0 while the minimum (12.5 mg/g) was observed at pH 9.0. Table 2 provides a comparison analysis of the adsorption of various dyes using chitosan powder and chitosan beads. Bekci¸ et al.^30^ investigated the effect of chitosan bead on the adsorption of the cationic dye Malachite Green. Similar to chitosan powder, the adsorption efficiency of chitosan beads increased with higher adsorbent doses, as more binding sites became available for dye molecules. The effect of solution pH was studied, and the results showed that chitosan beads could be suitable adsorbents at high pH ≥ 8 for cationic dye adsorption. The maximum adsorption capacities were 93.55 mg/g at pH 8 for 300 min. The study also stated that chitosan beads exhibited a higher saturation point compared to chitosan powder, implying that the optimal dose for chitosan beads might be higher before reaching its adsorption capacity limit.

**Table 2:** Comparison of the adsorption of different dyes using chitosan powder and chitosan beads.

Adsorbent	Dye	Adsorption capacity (mg/g)	Temperature (°C)	pH	Reference
Chitosan (powder)	Direct Blue 78	10.5	25 ± 2	6	This study
Chitosan (powder)	Acid black 1	18	30	7	^31^
Chitosan (powder)	Direct scarlet B	37.18	47.5	8.5	^32^
Chitosan (beads)	Direct Blue 78	6.9	25 ± 2	6	This study
Chitosan (beads)	Reactive black 5	4.83	25	7	^33^
Chitosan (beads)	Orange-G	79	30	8	^34^

## Adsorption isotherm

**Figure 8:**
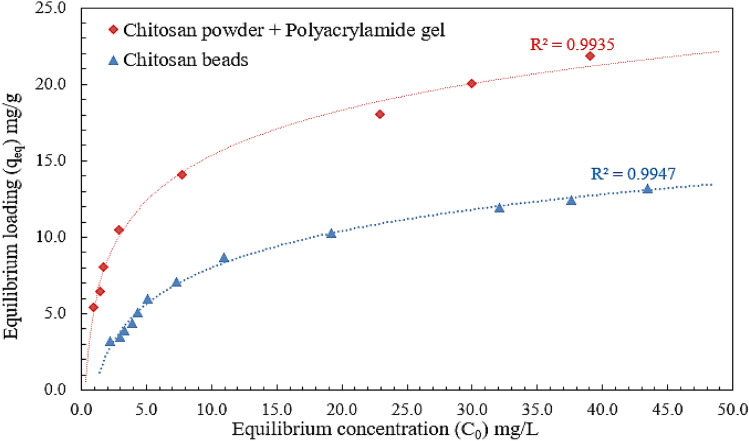
The adsorption isotherm for DB78 dye removal using chitosan powder and chitosan micro-beads.

The abstract discusses the absorption of DB78 dye by chitosan powder and chitosan micro-beads. The amount of dye absorbed by the adsorbents, known as QE, is directly proportional to the concentration of the dye in the liquid state. The shape of the adsorption isothermal curve in Fig. 8, is important to consider when designing adsorption systems, as it provides information on the equilibrium curve and other related phenomena. The equilibrium curves are divided into four groups based on their primary slope, with subgroups characterized by upper portion forms and slope changes. These groups include S curves or isotherms with vertical orientation, Langmuir isotherms or L curves, high-affinity isotherms or H curves, and C curves or isotherms of constant partition^35^.

The basic premise of the initial shape of the equilibrium curve (L shape) in Fig. 8 is that the higher the solute concentration, the greater the adsorption capacity until the number of adsorption site clearance is limited, resulting in competition between solute molecules for the available sites. Adsorption occurs due to relatively weak forces, such as "van der Waals forces," according to this isotherm type. There are several isothermal models (equations) available, and the two important isotherms, the Freundlich and Langmuir isotherms, were chosen for this study.

**Figure 9:**
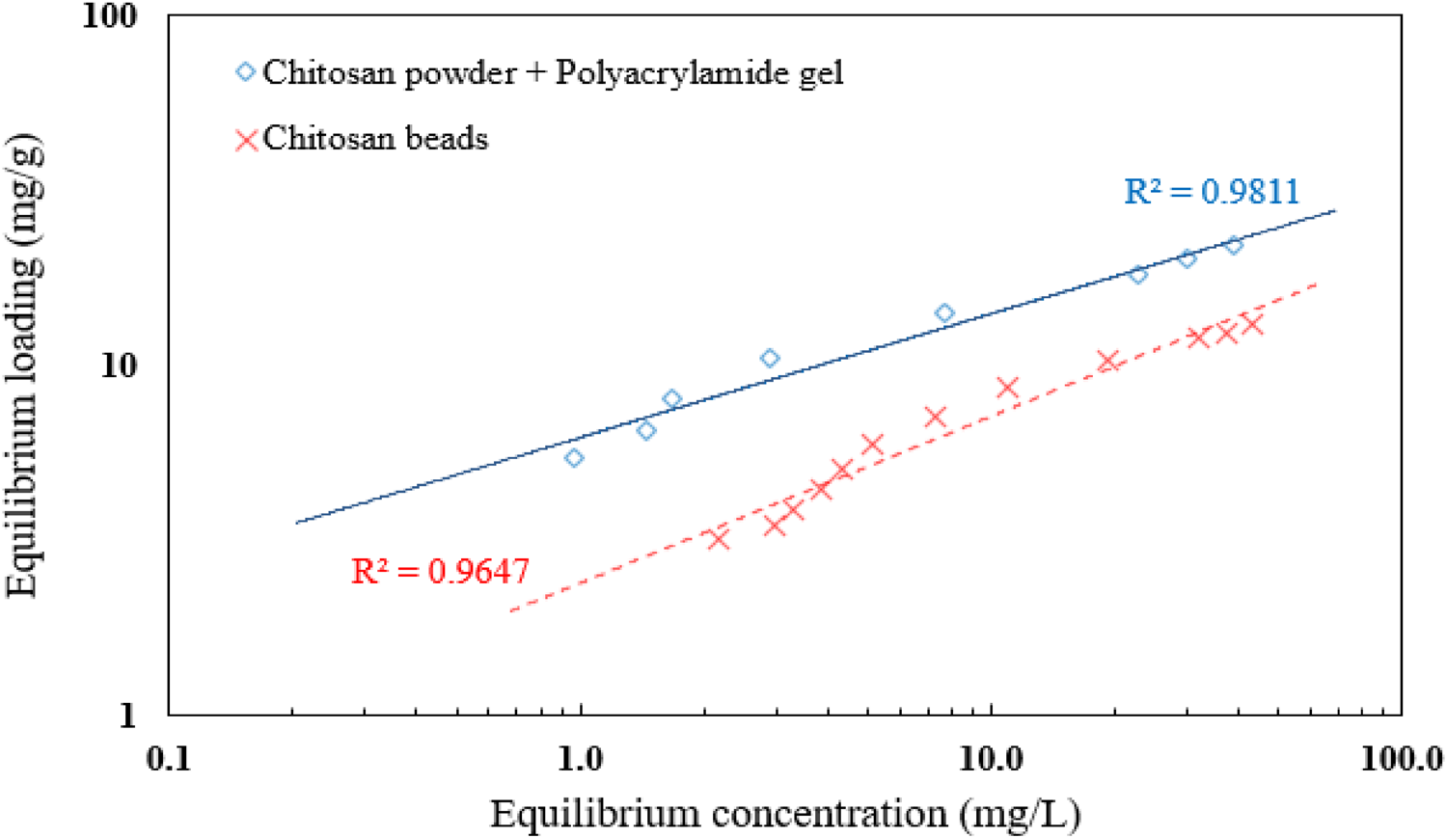
Freundlich isotherm for DB78 dye removal using chitosan powder and chitosan micro-beads.

The Freundlich isotherm believes that adsorption happens on a heterogeneous surface, and the adsorbed mass increases exponentially with an increase in concentration (Abraham et al. 2011). This isotherm explains equilibrium on heterogeneous surfaces and hence capacity is not presumed monolayer. In the liquid phase, this isotherm is given by (Eq. 3).

3 $${\text{Q}}_{{\text{e}}} = {\text{K}}_{{\text{F}}} {\text{C}}_{{\text{e}}}^{{{1}/{\text{n}}_{{\text{F}}} }}$$

where **Q**_**e**_ is the Freundlich fixed value (**K**_**F**_ unit = mg/g, and **C** = 1/n_F_ is the heterogeneity factor). This isotherm focuses on integrating the role of adsorbent–adsorbate surface interactions. Figure 9, indicates the application of equilibrium data according to the Freundlich isotherm. Chitosan powder as an adsorbent, the Freundlich constant **K**_**F**_ value was (6.22) and the heterogeneity factor 1/n_F_ value was (0.35) for the solution with an initial concentration of 50 mg/L. Using chitosan micro-beads as an adsorbent, the Freundlich constant **K**_**F**_ value was 2.39 mg/L and the heterogeneity factor 1/n_F_ value was 0.47 for an initial concentration of 50 mg/L.

The Langmuir isotherm holds that sorption occurs at different homogeneous sites within the adsorbent and has been successfully applied to several sorption processes. The isotherm's physical simplicity is based on some assumptions: (1) Adsorption cannot occur beyond monolayer coverage. (2) Each site can hold only one adsorbate molecule. (3) All sites are energetically equivalent and the surface is uniform.

**Figure 10:**
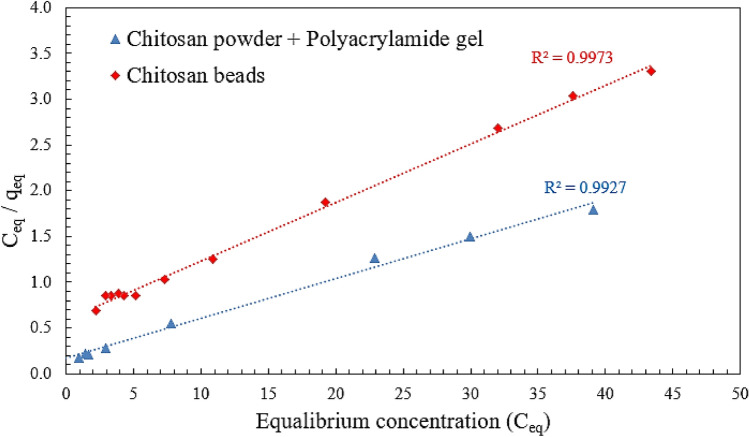
Langmuir isotherm for DB78 dye removal using chitosan powder/micro-beads.

The linear form of the Langmuir isotherm is given by the following equation (Eq. 4):

4 $$\left( {{\text{C}}_{{\text{e}}} /{\text{q}}_{{\text{e}}} } \right) = \left( {{1}/{\text{Q}}_{{\text{o}}} {\text{b}}} \right) + \left( {{\text{C}}_{{\text{e}}} /{\text{ Q}}_{{\text{o}}} } \right)$$

where **C**_**e**_ is the equilibrium concentration (mg/L), **he** is the mass adsorbed at equilibrium (mg/g), **Q**_**o**_ is the adsorbent loading (mg/g) and b is the adsorption energy (Langmuir fixed value l/mg). The values of **Q**_**o**_ and b were determined from the slope and intercept of the linear plots **C**_**e**_**/q**_**e**_ versus **C**_**e**_, resulting in a straight line of slope **1/Q**_**o**_ corresponding to the total coverage of Monolayer (mg/g) and the intercept is **1/Q**_**o**_** b** (Staroń, and Chwastowski 2021). Figure 10, indicates the application of equilibrium data according to the Langmuir isotherm. Using chitosan powder, the adsorbent loading value **Q**_**o**_ was 22.9 mg/g. The Langmuir isotherm can be used to calculate the separation factor (also known as the equilibrium parameter or dimensionless separation factor) represented by R_L_. The separation factor is an important indicator of the favorability of adsorption. The separation factor is defined as:

5 $${\text{R}}_{{\text{L}}} = {1}/({1} + {\text{K}}_{{\text{L}}} \cdot {\text{C}}_{0} )$$

where C_0_: is the initial concentration of the solute in the solution and K_L_: is the Langmuir constant related to the energy of adsorption. The results indicated that 0 ˂ R_L_ ˂1, indicates favorable adsorption. The adsorption capacity increases with increasing solute concentration, implying that the adsorption process is preferred.

Langmuir fixed value (b) was 0.26 1/mg for an initial concentration of 50 mg/L. Using chitosan micro-beads, the adsorbent loading value (Q_o_) was 15.65 mg/g, and Langmuir’s fixed value (b) value was 0.107 l/mg.

**Figure 11:**
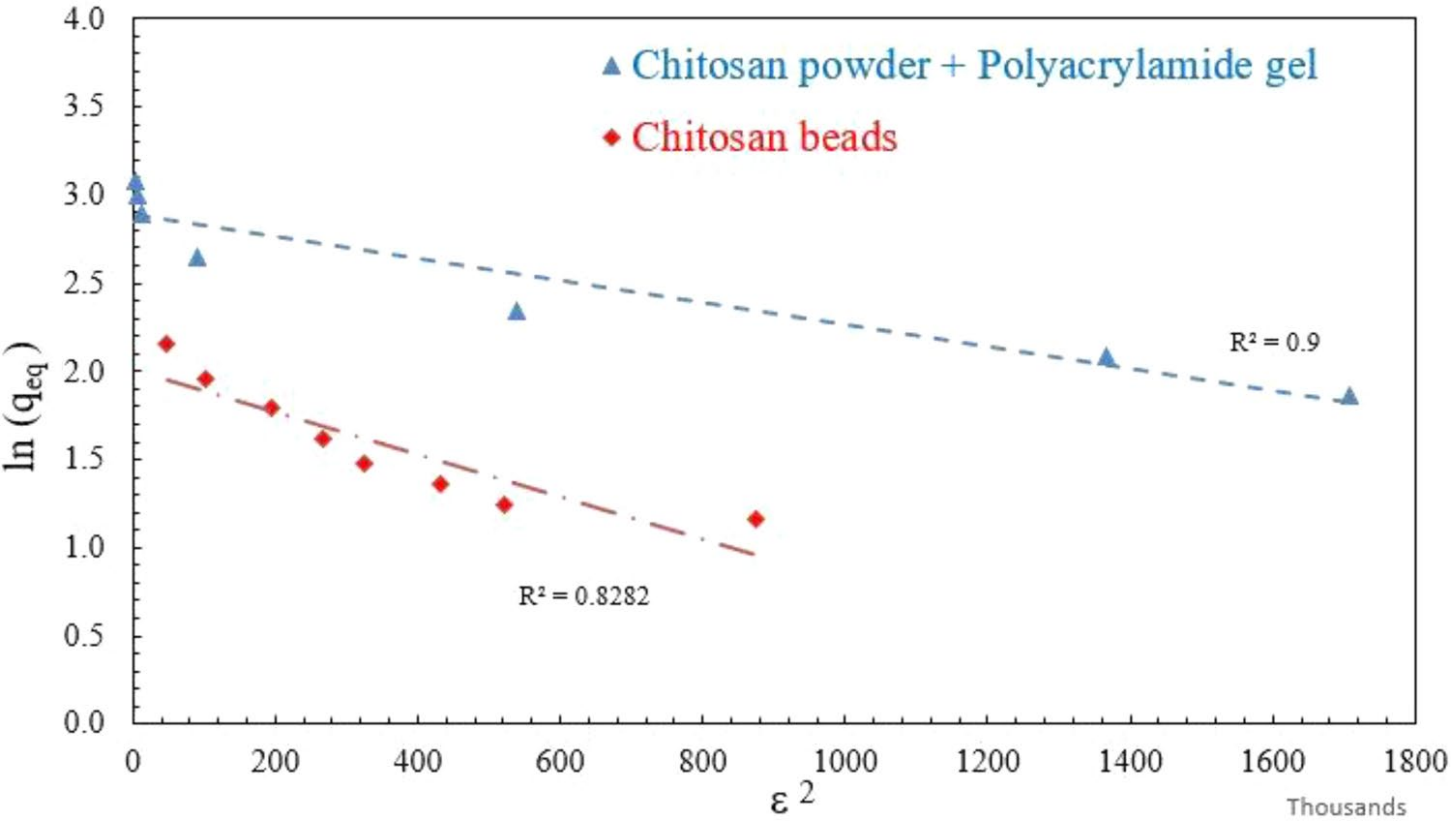
The Dubinin–Radushkevich (D–R) isotherm for DB78 dye removal using chitosan powder/micro-beads.

The Dubinin–Radushkevich (D–R) isotherm model is used to describe the physical adsorption of solutes on a heterogeneous surface. It is based on the assumption that the adsorption process occurs on a nonuniform surface with a distribution of adsorption energies. The model is particularly suitable for describing adsorption on porous materials with a wide range of pore sizes and adsorption energies ^36^. The isotherm is given by (Eq. 6).

6 $${\text{ln}}\left( {q_{{\text{e}}} } \right) = {\text{ln}}\left( {q_{{\text{m}}} } \right) - \beta \cdot \varepsilon^{{2}}$$

where *q*_e_: is the amount of solute adsorbed per unit mass of the adsorbent at equilibrium, q_m_: is the maximum adsorption capacity, *β*: is a constant related to the adsorption energy, and *ε:* is the Polanyi potential, which is related to the equilibrium concentration. Figure 11, indicates the application of equilibrium data according to the Dubinin–Radushkevich (D–R) isotherm.

The Harkins–Jura (H–J) isotherm considers the possibility of multilayer adsorption on adsorbents with heterogeneous pore distributions. In such cases, the surface of the adsorbent material may consist of different pore sizes and shapes, leading to the formation of multiple layers of adsorbed molecules, where A and B are the model constants^37^. The model can be expressed as shown in Eq. (4).

7 $$\left( {{1}/{\text{q}}_{{\text{e}}}^{2} } \right) = \left( {{\text{B}}/{\text{A}}} \right){-}\left( {{1}/{\text{A}}} \right){\text{logc}}_{{\text{e}}}$$

**Figure 12:**
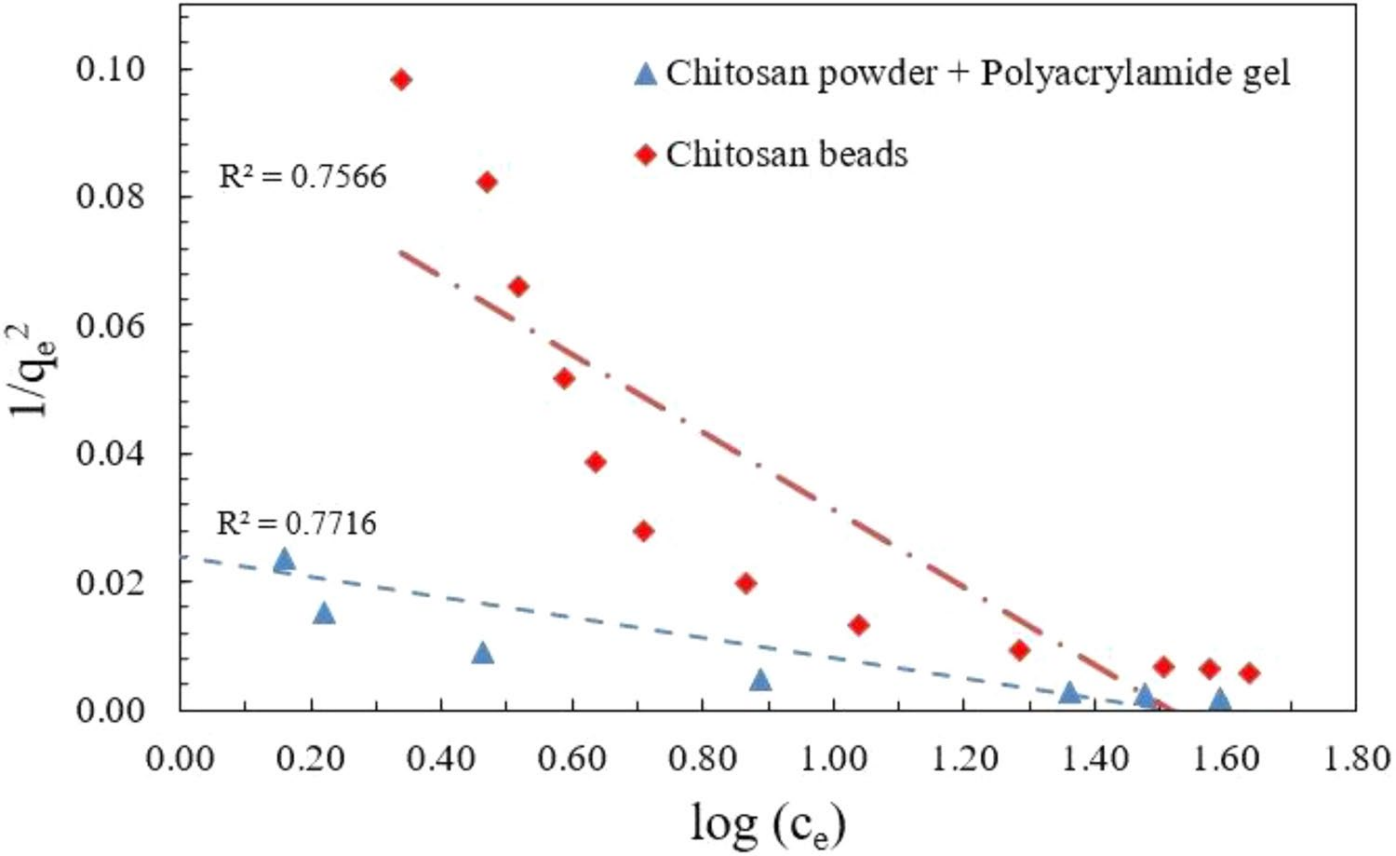
The Harkins–Jura (H–J) isotherm for DB78 dye removal using chitosan powder/micro-beads.

The Harkins–Jura isotherm’s inclusion of both a monolayer term (A term) and a multilayer term (B term) reflects this behavior. The A term represents the monolayer adsorption where adsorbate molecules form a single layer on accessible surface sites, while the B term accounts for the contribution of additional layers that might form on less accessible or smaller pores. Figure 12 showed that R-squared for chitosan beads and chitosan powder + polyacrylamide gels 0.7566 and 0.7716 respectively. This referred to a large amount of variability in the dependent variable that is not explained by the independent variable(s). This often results in data points scattered far from the regression line. It was observed from listed adsorption isothermal models in (Table 3) that the adsorption of DB78 dye using both neat chitosan powder and chitosan micro-beads follows Langmuir isotherm. Thus, the adsorption took place at specific sites on the surface and the adsorbed molecules did not interact with each other, and the adsorption process is reversible.

**Table 3:** Comparison of isothermal models for adsorption of different dyes using chitosan beads and chitosan powder.

Adsorbent	Dye	Langmuir isothermal		Freundlich isothermal		The Dubinin–Radushkevich		The Harkins–Jura isotherm		Model fitting	References
		Go (mg/g)	B (1/mg)	K_f_ (mg/g)	1/n	q_m_	β	A	B		
CS powder	Direct Blue 78	22.93	0.26	6.22	0.35	18	6 × 10–7	63.291	1.519	Langmuir	This study
CS powder	Remazol yellow 3RS	373	0.054	65.67	3.46	–	–	–	–	Langmuir	^34^
CS powder	Basic yellow 37	254	0.016	16.54	2.16	–	–	–	–	Langmuir	^34^
CS beads	Direct Blue 78	15.65	0.107	2.39	0.47	7.5	1 × 10–6	16.611	1.521	Langmuir	This study
CS beads	Remazol yellow 3RS	311	0.065	65.04	3.88	–	–	–	–	Langmuir	^34^
CS beads	Basic yellow 37	137	0.03	19.65	3.06	–	–	–	–	Langmuir	^34^

The Harkins–Jura isotherm's inclusion of both a monolayer term (A term) and a multilayer term (B term) reflects this behavior. The A term represents the monolayer adsorption where adsorbate molecules form a single layer on accessible surface sites, while the B term accounts for the contribution of additional layers that might form on less accessible or smaller pores. It was observed from listed adsorption isothermal models in (Table 3) that the adsorption of DB78 dye using both neat chitosan powder and chitosan micro-beads follows Langmuir isotherm. Thus, the adsorption took place at specific sites on the surface and the adsorbed molecules did not interact with each other, and the adsorption process is reversible.

**Figure 13:**
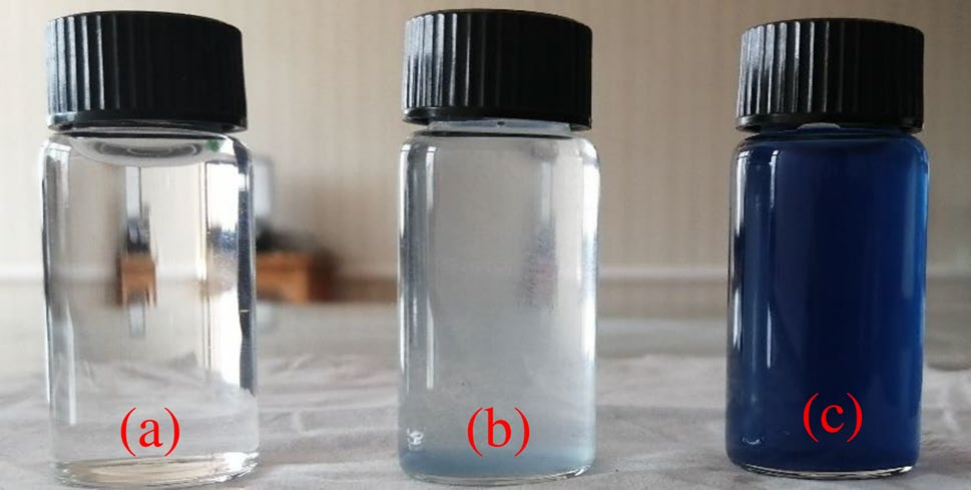
DB78 dye removal using: (**a**) Chitosan powder and (**b**) Chitosan micro-beads (**c**) Synthetic wastewater.

Figure 13 shows the synthetic wastewater, treated wastewater using chitosan powder, and treated wastewater using chitosan mico-beads. The two wastewater samples were treated using a fixed dose of 4.5 g/L for both chitosan powder and chitosan beads, the obtained removal efficiencies were 94.1% and 80% respectively. The synthetic wastewater was relatively stable and resistant to biodegradation as an indication to persist in the environment for a long time without degradation. The treated wastewater using chitosan powder and polyacrylamide gel is clear (the chitosan powder achieved a high efficiency for dye removal at 94.1% using a small dose of 4.5 g/L, while the polyacrylamide gel reduced the required settling time from 8 h to 5 min). In contrast, using the chitosan micro-beads to treat the synthetic wastewater with the same dose (4.5 g/L) showed a removal efficiency of 80% with instantaneous sedimentation without using polyacrylamide gel.

### Adsorption kinetics

**Figure 14:**
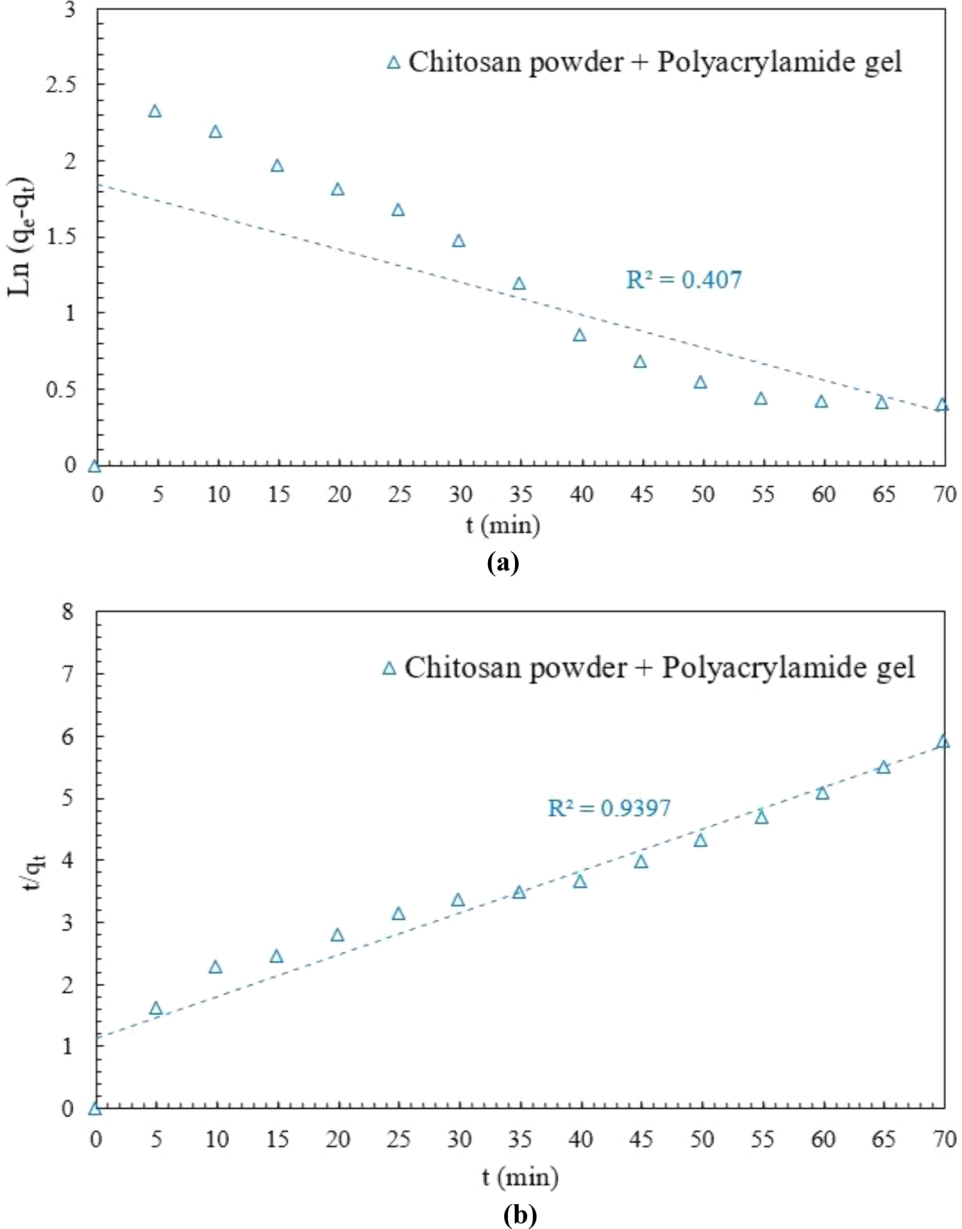
Adsorption kinetic studies (**a**) pseudo-first-order model. (**b**) pseudo-second-order model.

To understand the mechanism of the adsorption process, the kinetic studies were conducted by extracting and analyzing the samples at time intervals of 5 min until the consecutive residue dye concentrations became closer. The kinetic data for the adsorption process of DB78 dye onto chitosan powder, with initial dye con-centration 50 mg/L were examined with the well-known kinetic models namely the pseudo-first-order model (PFO) and pseudo-second-order model (PSO). The plotting of this kinetic model is shown in Fig. 14.

Pseudo first-order equation:

The pseudo-first-order kinetic equation was used for adsorption analysis. The linear form of this equation is

8 $${\text{ln}}\left( {{\text{qe}} - {\text{qt}}} \right) = {\text{lnqe}}{-}{\text{k}}_{{1}} {\text{t}}$$

where qe (mg/g) and qt (mg/g) are the amounts of adsorbed adsorbate at equilibrium and at time t, respectively. K_1_ (min^-1^) is the rate constant of the pseudo-first-order model.

Pseudo second-order equation:

The adsorption kinetics can also be described by a pseudo-second-order model. The linear form of the pseudo-second-order equation is expressed as

9 $$\left( {{\text{t}}/{\text{qt}}} \right) = \left( {{1}/{\text{k}}_{{2}} {\text{q}}_{{\text{e}}} {2}} \right) + \left( {{1}/{\text{q}}_{{\text{e}}} } \right){\text{t}}$$

where k_2_ (g/mg min) is the equilibrium rate constant of pseudo-second-order adsorption. where q_e_ (mg/g) and qt (mg/g) are the amounts of adsorbed adsorbate at equilibrium and at time t, respectively ^38^.

Figure 14 shows the linear plots of PFO and PSO models of chitosan powder. Based on the low correlation coefficient for PFO (R^2^ = 0.407) and the high value for PSO (R^2^ = 0.939), the adsorption abilities of chitosan powder follow PSO rather than PFO. For chitosan, the applicability of the PSO model indicates the interaction between dye molecules and amino groups. Hence, the adsorption system is chemical adsorption. It was reported that the adsorption process of DB78 dye onto chitosan is best fitted to pseudo-second order with a chemical adsorption mechanism^39^.

## Conclusions

Separation and removal of direct blue 78 dye from synthetic solutions by adsorption with powder chitosan with the aid of polyacrylamide gel and chitosan beads were experimentally examined. The experiments were carried out using synthetic wastewater applied on the batch scale. In this study, it was found that (1) The dye removal efficiency increased with increasing chitosan dosage and contact time at low pH; (2) Experimental studies showed that the maximum capacities for dye removal were 94.4% and 94.1% for initial con centra tion 50 mg/l using adsorbent dose 4.5 g/L and 13.5 g/L for powder chitosan and chitosan beads respectively. In adsorption studies, it was found that polyacrylamide gel accelerated the settling time of chitosan. It decreased the settling time from 8 h to 5 min. The maximum obtained adsorption capacities of chitosan (powder) and chitosan (beads) were 10.5 mg/g and 26.3 mg/g respectively. Equilibrium studies showed that the initial shape of the equilibrium curve (L shape) which means that the adsorption process resulted from electrostatic interaction between dye molecules and adsorbent particles (physical forces). It was observed that the Langmuir isothermal model best suited the equilibrium results. Chitosan powder combined with polyacrylamide gel can be used as an affordable option to remove dyes from industrial wastewater effluents, considering the high cost of commercial adsorbents.

## Data Availability

The datasets used and/or analyzed during the current study are available from the corresponding author upon reasonable request.
